# Space Availability in Confined Sheep during Pregnancy, Effects in Movement Patterns and Use of Space

**DOI:** 10.1371/journal.pone.0094767

**Published:** 2014-04-14

**Authors:** Xavier Averós, Areta Lorea, Ignacia Beltrán de Heredia, Josune Arranz, Roberto Ruiz, Inma Estevez

**Affiliations:** 1 Department of Animal Production, Neiker-Tecnalia, Vitoria-Gasteiz, Spain; 2 Navarra Public University (UPNA), Pamplona, Spain; 3 IKERBASQUE, Basque Foundation for Science, Bilbao, Spain; Institut Pluridisciplinaire Hubert Curien, France

## Abstract

Space availability is essential to grant the welfare of animals. To determine the effect of space availability on movement and space use in pregnant ewes (*Ovis aries*), 54 individuals were studied during the last 11 weeks of gestation. Three treatments were tested (1, 2, and 3 m^2^/ewe; 6 ewes/group). Ewes' positions were collected for 15 minutes using continuous scan samplings two days/week. Total and net distance, net/total distance ratio, maximum and minimum step length, movement activity, angular dispersion, nearest, furthest and mean neighbour distance, peripheral location ratio, and corrected peripheral location ratio were calculated. Restriction in space availability resulted in smaller total travelled distance, net to total distance ratio, maximum step length, and angular dispersion but higher movement activity at 1 m^2^/ewe as compared to 2 and 3 m^2^/ewe (P<0.01). On the other hand, nearest and furthest neighbour distances increased from 1 to 3 m^2^/ewe (P<0.001). Largest total distance, maximum and minimum step length, and movement activity, as well as lowest net/total distance ratio and angular dispersion were observed during the first weeks (P<0.05) while inter-individual distances increased through gestation. Results indicate that movement patterns and space use in ewes were clearly restricted by limitations of space availability to 1 m^2^/ewe. This reflected in shorter, more sinuous trajectories composed of shorter steps, lower inter-individual distances and higher movement activity potentially linked with higher restlessness levels. On the contrary, differences between 2 and 3 m^2^/ewe, for most variables indicate that increasing space availability from 2 to 3 m^2^/ewe would appear to have limited benefits, reflected mostly in a further increment in the inter-individual distances among group members. No major variations in spatial requirements were detected through gestation, except for slight increments in inter-individual distances and an initial adaptation period, with ewes being restless and highly motivated to explore their new environment.

## Introduction

Husbandry practices in domestic sheep (*Ovis aries*) can differ greatly, ranging from eminently extensive pasture, to intensive, indoor systems [1], differing greatly in the quantity and quality of space [2], which will largely determine sheep behaviour [3]. Sufficient space availability is considered to be essential to assure the health and welfare of production species [4] and is considered as one of the ‘Five Freedoms’ [5].

In production species, a reduction in the space provided to growing-finishing pigs will alter their resting activity and exploratory, social, and feeding behaviours [6]–[8], and will increase the occurrence of aggressive behaviour [9], resulting in reduced performance [10]. Resting, aggressive interactions, and performance in cattle [11]–[13], as well as the frequency of behavioural disturbances, lesion prevalence, and performance in poultry [14]–[17], are known to increase by decreasing space availability. In pregnant ewes, low space availability is associated with decreased activity, increased occurrence of social interactions [18], and changes in resting patterns [19]. It is also associated with reduced air microbial quality, milk hygiene, and higher prevalence of mastitis in lactating ewes [20], and with compromised feed efficiency and normal growth of young lambs [21]–[23]. These studies support the idea that a reduction in the space availability will translate into a reduction in welfare [24]. However, once space requirements have been met, further increments will not *per se* lead to improved welfare [25], since the adequacy of space may differ according to aspects such as the quality of space, the presence of environmental enrichment [7], individual or group housing [26], and to animal-related aspects such as familiarity, breed, and size of animals.

Domestic sheep form small to moderate social groups [27]. Movement and space use depend on the amount of space, being in itself a basic resource that may also be influenced by environmental complexity [15], resource availability and location, the presence of familiarity clues [28], and social behavioural components. Therefore, movement patterns are strongly influenced by neighbouring flock mates [29]–[31] and attractive and repulsive social forces modulating inter-individual distances [32]–[34]. This interrelationship explains why reductions in the space availability can have such remarkable effects on social patterns [35], hindering the free movement of individuals within the group because of the closer presence of other conspecifics [36]. The movement patterns and spatial distribution of animals have been proposed as a more sensitive measure of space requirements [37], resulting in valuable information about the potential consequences of restriction in the physical environment of sheep under confined conditions, and in a deeper evaluation of ovine production systems from an animal welfare perspective.

Previous studies have shown that enclosure size has a stronger effect on travelled distances and dispersion than group size or density in poultry [38], [39], although stocking density modulates this effect [31]. Interaction of group size and feeders' distribution on inter-individual distances [30], or higher use of enclosure peripheral areas at higher stocking densities, have also been reported in chickens [40], [41]. Use of space of rabbits depends on their behaviour [42], while inter-individual distances in resting growing-finishing pigs have been related to their growth, activity and feeding behaviour [43].

In sheep, previous studies have focused on their use of space in natural pastures [44]–[46] but no study has examined the spatial requirements of ewes under confined conditions, which are more likely to occur during pregnancy in order to protect them for harsh weather conditions. Tolerance for close inter-individual-distances, movement and use of space is generally higher in selected breeds [3], with females being more reactive than males to sources of stress [47], so that stress due to spatial limitation might be particularly detrimental for pregnant ewes. Pregnancy in ewes lasts for about 147 days, although gestation in Latxa breed, raised in Northern Spain for dairy purposes, is longer (about 154 days). In the UK, legal minimum space allowance for ewes varies from 1 to 2.2 m^2^/ewe (www.gov.uk/sheep-and-goat-welfare), and the European Council Regulation (EEC) 2092/91 on Organic Production states that ewes housed indoor must be provided with at least 1.5 m^2^/animal. But spatial requirements for pregnant ewes may also vary throughout gestation, with space restriction further compromising their movements and the use of space.

The aim of this study was to determine the effect of space availability on movement patterns and use of space during the second half of the gestation period in pregnant ewes. Previously published results of the behavioural aspects of this study [18] have shown that spatial restriction affects the time spent moving and the amount of social interaction, so that it is hypothesized that severe spatial restriction will also hinder the movements and use of space of gestating ewes, with this effect becoming gradually more evident as gestation progresses. Results from this study may have direct implications on the management of flocks kept under intensive conditions, particularly from the point of view of the amount of space ewes are provided with, and how this relates to their welfare.

## Materials and Methods

The experiment was approved by the NEIKER-Tecnalia Animal Experimentation Committee (Reference AFA_2011_02), and was carried out according to the European Directive 86/609/ECC regarding the protection of animals used for experimental and other scientific purposes. The experiment was designed to detect differences in welfare indicators of pregnant ewes associated to different space availability during pregnancy. Ewes were monitored throughout the whole experiment, and no major animal health and welfare issues, directly attributable to treatments, were observed.

### Facilities and experimental animals

This study was conducted at the experimental dairy sheep farm of Neiker-Tecnalia (Arkaute, Spain) between August 2011 and January 2012. The studied ewes (*Ovis aries*), and the rest of the Neiker-Tecnalia flock, belong to the Latxa breed. This breed is raised in Northern Spain for dairy production reasons, and adult ewes weight about 55 kg. Ewes were managed as a single experimental flock until the beginning of the experiment. Further details about general animal management are provided in a separate paper [18].

### Experimental design

All the ewes were artificially inseminated (AI) at the end of August. Forty-six days after AI, gestation and number of viable foetuses were confirmed via ultra-sound methodologies (Ovi-scan 6, BCF, Australia) and body condition was determined using a 5-point scoring scale [48]. Among those with confirmed pregnancy, 54 one to five year old ewes were randomly selected for the experiment, and were sheared at that moment. Sixty-two days after AI selected ewes were weighed and divided into nine groups of six individuals. Groups were balanced for body condition score, age and number of viable foetuses and assigned to one of the three space availability treatments (1, 2, or 3 m^2^/ewe) at a constant group size of 6 ewes/enclosure, with three replicates per treatment. All enclosures had solid PVC walls to prevent visual contact of the animal, and pen dimensions were 2.7×2.25, 2.7×4.5 and 3×5.9 m, respectively. Ewes were maintained in these enclosures until the end of the experimental period, one month after parturition. All animals were marked on the back area for individual recognition (purple spray, Multi-line, Ukal, France).

Feed was provided in an automatic feeding line, with eight individual feeding spaces (48 cm/ewe) per enclosure allowing simultaneous access to feed to all animals. From the beginning of the study, at the end of week 8 through to gestation week 15 ewes were feed silage twice/day, at 08:30 and at 15:00 (about 1.5 kg in total per ewe and day), while from 15 to 18 weeks fescue hay was provided twice/day at the same time (about 1.5 kg in total per ewe and day). The diet was complemented with 400–500 g of a barley and wheat mix/ewe in the morning meal, and with *ad libitum* access to oat hay and peas in the afternoon meal. From week 18 to 20 fescue hay was provided twice/day (about 1.5 kg in total per ewe and day), complemented with 500 g of concentrate (1.101 UFL/kg; 168 g PB/kg) per ewe in the morning meal. From week 12 to 16 pregnant ewes had free access to salt blocks (TIMAC SAS, St Malo, France) after which time blocks were substituted by a cube containing vitamin-mineral corrector (INAFORM, Timac Agro, Orcoyen, Spain). Drinking water was available *ad libitum* through an automatic drinking nipple installed in each enclosure. Straw bedding was provided throughout the whole experiment, and fresh straw was periodically added to maintain the bedding in good condition.

### Data collection

Data collection started in gestation week 9 (69 days after AI), 7 days after ewes were housed in the experimental pens, and lasted for 11 weeks (end of gestation week 19). To achieve a precise visual location of the individuals during the observations, the enclosures were divided into a visual grid (25, 45, and 72 squares for 1, 2, and 3 m^2^/ewe respectively), by placing numerical and alphabetical stickers along the walls of the enclosures according to the enclosure dimensions, following what has been previously described [30].

Observations started about 09:30, after the morning meal, and were conducted in two rounds per day, two days per week. During each round, each enclosure was observed for 15 minutes by continuous scan sampling. The order in which enclosures were observed was random for each round. Within each scan the withers position (in XY coordinates, obtained with the visual help of the stickers that defined a pen grid) and the behaviour of all ewes were sequentially collected using the Chickitizer software [49], a computer application specifically developed for the collection of spatial location and behaviour data simultaneously. This software allows data collection on a graphic representation of the experimental enclosure with a simple mouse click. To achieve this, real measures of the enclosure were previously defined and the data entry screen was customized according to such measurements. Averages of 12 scans/enclosure were collected per 15 minute samplings (i.e., for each sampling, an average of 12 XY values were collected per ewe). Results of behavioural observations have been reported elsewhere [18]. One ewe from the lowest space allowance treatment died during week 9 of the experiment due to complications derived from a uterine prolapse. Since the experiment was close to the end, and to avoid the social disruption that may be caused by the introduction of a new individual in the already stable social group, it was decided not to replace the dead animal and to continue the data collection regularly.

During data collection, clicking on an *a priori* identical position unavoidably results in slightly minor different XY values. The error was too small to alter in any way the results of the defined use of space and movement parameters. However, movement activity was defined as the change in position of the animal in two consecutive scans. Therefore, although the error was minimal it scored as a change in position that did not actually occur. Therefore, to eliminate this problem, an estimation of the error made during data collection was calculated for each space availability treatment. This was done for each enclosure size by clicking 20 times on *a priori* identical position with the Chickitizer. Then mean XY values of these positions were calculated, with the error being assumed to be the longest distance from among those obtained. Once the error for each enclosure size was estimated, the resulting ewes' position was corrected by evaluating if the distance between two subsequent scans was smaller than the estimated error. If this distance was smaller, it was assumed that the ewe did actually not move.

From corrected XY locations, a series of parameters to characterize the movement trajectories and space use of ewes were calculated [30], [50], [51]. Variables included total and net distance, net to total distance ratio, maximum and minimum step length, angular dispersion, movement activity, mean inter-individual, nearest neighbour and furthest neighbour distance. Additionally, the use of wall space relative to the total space in the enclosure was estimated. To do this, the peripheral enclosure area was defined as the area covering the 50 cm closer to the wall, a value slightly larger than a ewe's average width [19]. Then a peripheral location ratio and a corrected peripheral location ratio were calculated. Further details about variables and how they are calculated are included in Table 1.

**Table 1:** Variables measured in the study.

Variable	Definition
Total Distance (cm)	Total distance = 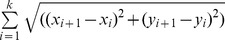 , that is, the sum of the euclidean distances between the k consecutive locations composing the trajectory of one ewe during one observation period.
Net Distance (cm)	Net distance = 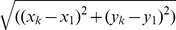 , that is, the euclidean distance between first and last location of the trajectory of one ewe during one observation period.
Net to Total distance ratio	Ratio between Net to Total distances.
Mean inter-individual distance (cm)	Within the same scan, mean Euclidean distance between the locations of all the ewes within a pen.
Minimum step length (cm)	Shortest Euclidean distance between two of the locations composing the trajectory of one ewe during one observation period.
Maximum step length (cm)	Longest Euclidean distance between two of the locations composing the trajectory of one ewe during one observation period.
Nearest neighbour distance (cm)	Within the same scan, Euclidean distance between the location of a given ewe and that of the closest ewe within the pen.
Furthest neighbour distance (cm)	Within the same scan, Euclidean distance between the location of a given ewe and that of the furthest ewe within the pen.
Angular dispersion	For the k consecutive locations composing the trajectory of one ewe during one observation period, angular dispersion = 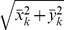 , where 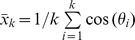 and 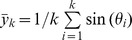 , being θi the turning angle between the ith location and the (i-1)th location.
Movement activity	Frequency of scans in which the position of a ewe differed from that in the previous scan.
Peripheral location ratio	Frequency of scans in which a ewe was located in the peripheral area, defined as the area covering the 50 cm closer to the wall.
Corrected peripheral location ratio	Peripheral ratio minus the expected peripheral location ratio value if the ewes were located at random within the enclosure.

### Statistical analysis

For all the dependent variables, mean values were calculated per enclosure and week. Normality and variance homoscedasticity of data were tested and confirmed. The effects of space availability, the experimental week, and their two-way interaction on the average enclosure values of all dependent variables were tested by means of a repeated measures mixed model ANOVA, with the gestation week being used as the repeated measures unit, and with the enclosure being included as a random factor in the model. Least square means were computed in case of statistically significant effects (*P*<0.05), with *P*-values adjusted for multiple comparisons by Tukey range tests. All statistics were performed using SAS 9.3 (SAS Institute, Cary, NC, USA).

## Results

Space availability and week had a significant effect over most of the studied movement and use of space parameters, but none of the interactions were significant (Table 2).

**Table 2:** Results for the mixed model ANOVA for the effects of space availability, week, and their interaction on the movement and use of space parameters.

Variables	Space availability		Week		Space availability × Week	
	F_2,6_	*P*	F_10,60_	*P*	F_20,60_	*P*
Total distance	9.27	0.0146	6.18	<0.0001	1.30	0.2134
Net distance	4.55	0.0627	2.49	0.0142	1.42	0.1489
Net to total distance ratio	29.14	0.0008	5.27	<0.0001	1.50	0.1146
Maximum step length	18.65	0.0027	3.88	0.0004	1.25	0.2464
Minimum step length	2.63	0.1513	15.66	<0.0001	1.22	0.2679
Mean Inter-individual distance	45.31	0.0002	1.55	0.1435	0.90	0.5869
Nearest neighbour distance	44.04	0.0003	2.53	0.0130	1.32	0.2053
Furthest neighbour distance	72.48	<0.0001	1.22	0.2943	0.72	0.7886
Peripheral location ratio	10.42	0.0112	1.89	0.0640	1.10	0.3729
Corrected Peripheral location ratio^1^	1.66	0.2664	1.89	0.0640	1.10	0.3729
Movement activity	19.62	0.0023	8.60	<0.0001	1.39	0.1629
Angular dispersion	17.09	0.0033	4.11	0.0002	0.80	0.7078

1 : Observed – Expected values.

**Figure 1:**
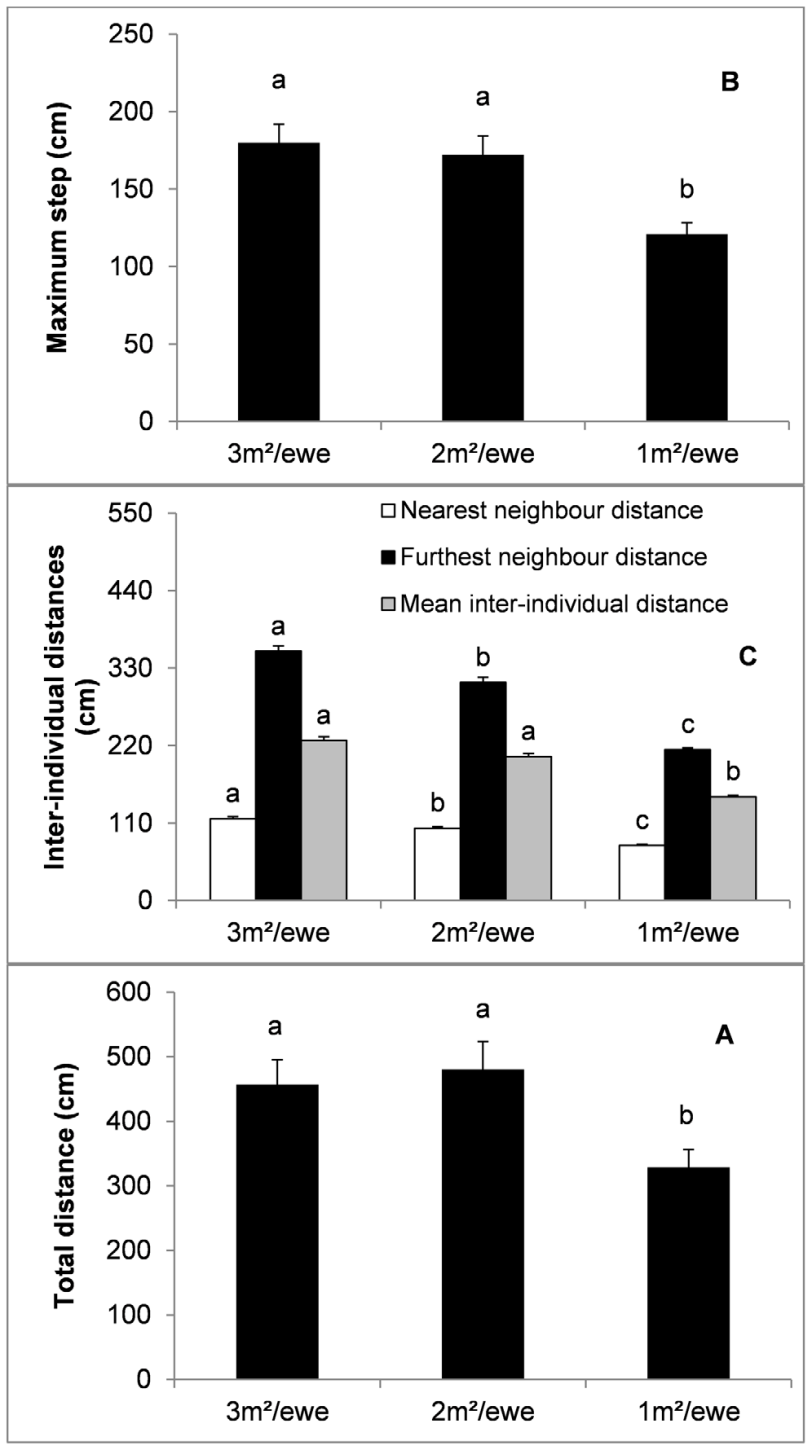
Effect of space availability (mean ± SE) on total travel distance (A), maximum step length (B) and inter-individual distances (C). Within each variable, different letters indicate statistically significant differences (*P*<0.05).

Reduced space availability had an important effect on the parameters that described ewes' movement patterns. The limitation of space availability became apparent on total travelled distance, (*P*<0.05; Fig. 1A) and maximum step length (*P*<0.01; Fig. 1B), being lower at 1 m^2^/ewe as compared with 2 and 3 m^2^/ewe. A tendency for shorter net distances (93.4±5.7 cm, 128.3±8.1 cm, and 127.6±9.0 cm for 1, 2 and 3 m^2^/ewe respectively; *P*<0.10) was observed, while no significant differences were detected for minimum step length. Lower space availability also determined shorter mean neighbour distances compared to 2 and 3 m^2^/ewe, while nearest and furthest neighbour distances increased progressively form 1 to 3 m^2^/ewe (*P*<0.001; Fig. 1C)

**Figure 2:**
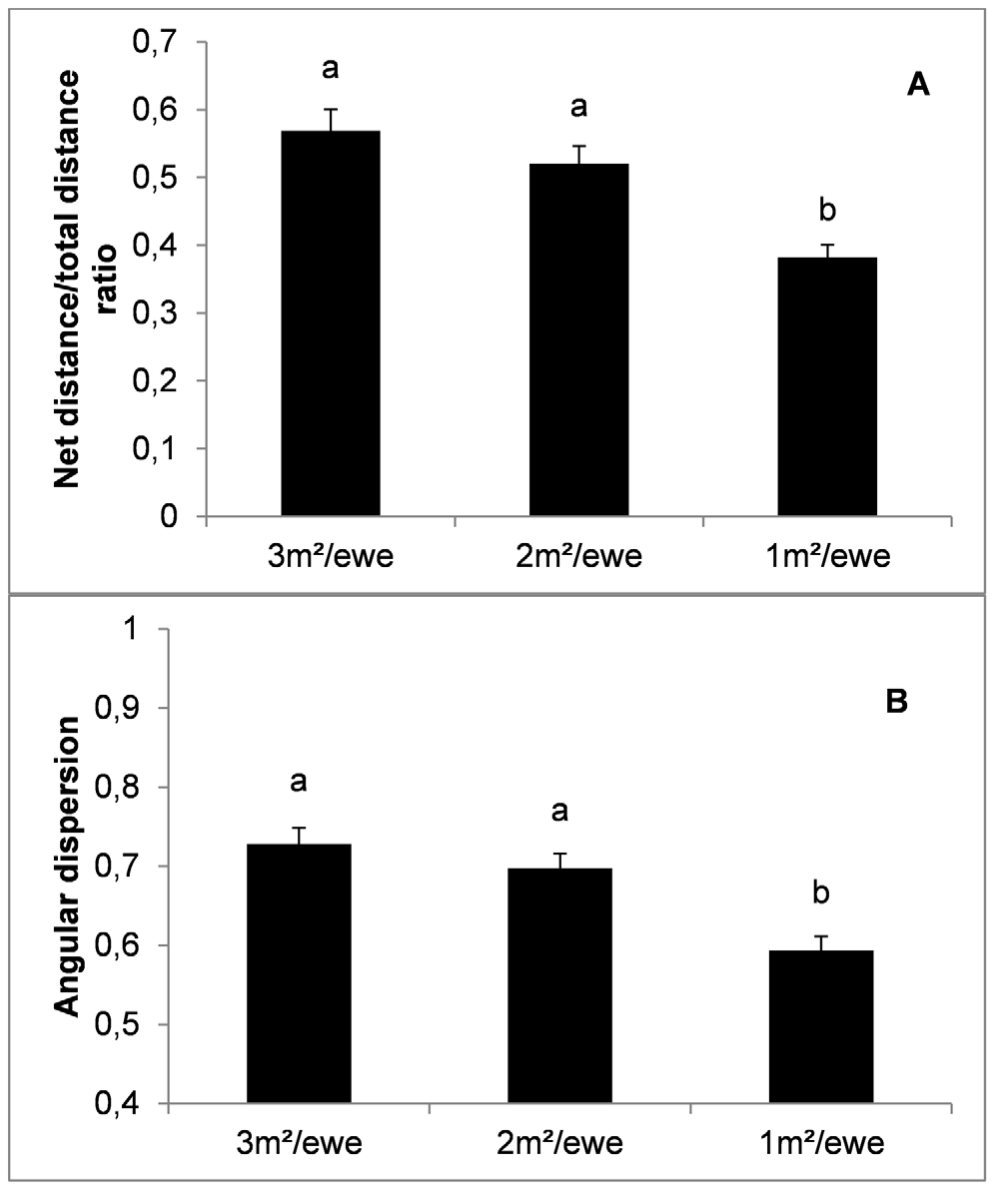
Effect of space availability (mean ± SE) on net to total distance ratio (A) and angular dispersion (B). Different letters indicate statistically significant differences (*P*<0.05).

**Figure 3:**
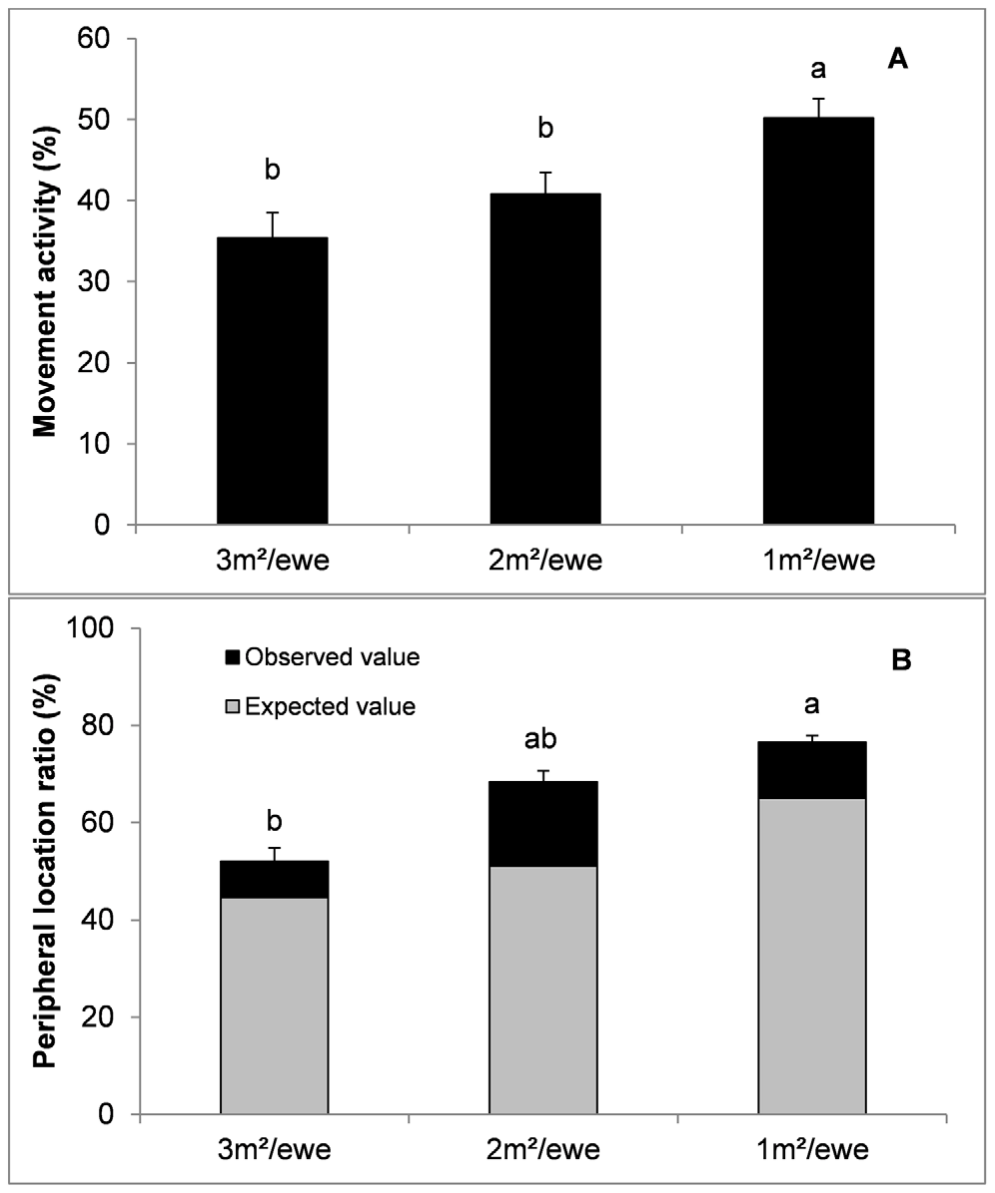
Effect of space availability (mean ± SE) on movement activity (A) and peripheral location ratio (B). Different letters indicate statistically significant differences (*P*<0.05).

Net to total distance ratio (*P*<0.001; Fig. 2A) and angular dispersion (*P*<0.01; Fig. 2B) were lower at 1 m^2^/ewe as compared to 2 and 3 m^2^/ewe. Lower space availability also determined a higher rate of movement activity (*P*<0.01; Fig. 3A) and a higher use of the peripheral area (*P*<0.05; Fig. 3B). However, statistical differences regarding the use of the peripheral areas vanished when the use of the peripheral area was corrected according to a random distribution of ewes within the pen (see Table 2 for corrected peripheral location ratio).

In regard to the effects of experimental weeks (see Table 2) differences detected in total travelled distance, net to total distance ratio, maximum and minimum step length, and movement activity (*P*<0.001; Table 3) were mainly due to differences observed during the first week of the study, when travelled distances were generally the longest, and the net to total distance ratio was the smallest. Similarly, angular dispersion was lower during the first week of study (*P*<0.001), with values remaining higher during the remaining weeks of the study, except for weeks 6 and 8, where values did not statistically differ from the initial ones. Nearest neighbour distance values showed a slight increase over the gestation period from the first to the last week of observation (*P*<0.05; Table 3).

**Table 3:** Changes in the studied variables (mean ±SE) along the experimental weeks.

	Experimental week																					
	1 (n = 9)		2 (n = 9)		3 (n = 9)		4 (n = 9)		5 (n = 9)		6 (n = 9)		7 (n = 9)		8 (n = 9)		9 (n = 9)		10 (n = 9)		11 (n = 9)	
	Mean	SE	Mean	SE	Mean	SE	Mean	SE	Mean	SE	Mean	SE	Mean	SE	Mean	SE	Mean	SE	Mean	SE	Mean	SE
Total distance (cm)	782a	82	316b	57	380b	34	470b	62	469b	89	483b	52	320b	63	419b	65	254b	55	383b	42	368b	56
Net distance (cm)	142a	10	87ab	16	134ab	14	137a	17	119ab	14	128ab	11	107ab	18	123ab	19	75b	14	110ab	14	118ab	16
Net to total distance ratio	0.26b	0.02	0.54a	0.05	0.61a	0.04	0.45a	0.05	0.47a	0.04	0.42ab	0.06	0.54a	0.07	0.51a	0.05	0.51a	0.05	0.53a	0.05	0.55a	0.05
Maximum step length (cm)	243a	25	129b	24	162ab	11	169ab	20	166ab	22	176ab	18	119b	18	164ab	24	114b	22	150b	18	143b	17
Minimum step length (cm)	11.85a	2.43	1.63b	0.76	0.69b	0.36	1.27b	0.59	2.31b	1.44	0.36b	0.29	0.03b	0.02	0.63b	0.46	0.69b	0.46	0.95b	0.53	0.12b	0.12
Mean inter-individual Distance (cm)	173	12	182	16	200	14	194	13	191	14	187	11	192	16	202	17	195	13	200	14	206	15
Nearest neighbour distance (cm)	86a	5	94ab	7	99ab	6	100ab	8	97ab	7	93ab	5	99ab	8	106ab	8	99ab	7	104ab	6	111b	8
Furthest neighbour distance (cm)	267	21	277	25	303	24	295	22	290	22	279	20	301	28	304	27	296	20	297	23	309	25
Peripheral location ratio (%)	60.13	6.68	61.57	4.47	76.00	6.13	67.07	6.51	64.46	8.03	64.91	5.82	69.67	3.94	71.78	3.40	60.38	4.34	62.05	4.76	64.00	3.26
Corrected Peripheral location ratio^1^ (%)	6.57	4.97	8.01	4.58	22.44	5.11	13.51	5.43	10.91	6.55	11.35	3.70	16.11	4.11	18.22	2.72	6.82	3.17	8.49	3.00	10.44	1.04
Movement activity (%)	73.34a	2.66	36.28b	4.84	38.52b	3.62	44.09b	5.15	42.11b	2.98	43.27b	4.80	38.08b	4.55	41.88b	5.18	28.87b	3.52	39.71b	5.01	37.46b	5.61
Angular dispersion	0.50b	0.02	0.72a	0.05	0.70a	0.03	0.66a	0.04	0.68a	0.02	0.64ab	0.06	0.71a	0.04	0.65ab	0.04	0.75a	0.03	0.67a	0.04	0.71a	0.04

Within each row, different letters (a–e) indicate statistically significant differences (*P*<0.05).

1 Observed – Expected.

## Discussion

### Space availability

Sufficient space availability is essential to ensure the welfare of confined animals, which should be estimated not only by considering their behavioural needs but also the patterns of movement and space use. In this study it was hypothesised that a reduction in the space availability from 3 to 1 m^2^/ewe would result in a restriction in the movement patterns and use of space, as evidenced by shorter, more sinuous trajectories and reduced inter-individual distances. It was also predicted that the effects of spatial restriction would become more evident as gestation advanced, as a consequence of the increment in body size. The results obtained in this study confirm this hypothesis, as most considered parameters were clearly affected by space treatment. Total travelled distance, maximum step length, and nearest and furthest neighbour distances were significantly shorter, while movement activity increased when space was restricted to 1 m^2^/ewe as compared to 2 and 3 m^2^/ewe. In addition, movement trajectories were more sinuous at the lowest space availability treatment, as indicated by the lower values obtained for the net to total distance ratio and angular dispersion. Movement activity, a parameter that estimates the rate of disturbances [31] was also higher.

In this study space availability was altered while keeping group size constant to control for social effects on movement patterns, and the only possible way to accomplish this was by changing enclosure size. As a result, the effects of space availability per individual (density related effects), and those of the enclosure size were confounded. An alternative would have been to maintain enclosure size constant while changing group size, but this would have led to other confounding effects [31]. Hence, given the experimental constrains it is difficult to specifically determine whether the observed restriction in movements, the changes in the sinuosity of the ewes' trajectories, and the higher movement activity were related to the small size of the enclosure, to density related effects, or to a combination of both. Nevertheless, while in a small enclosure individuals should ‘in principle’ be able to move around, even if forced to follow more sinuous movement trajectories as compared with larger enclosures, and could potentially yield to similar total distance travelled. However, as indicated, total distance travelled was significantly shorter, and net to total distance ratios and angular dispersion values significantly lower for space availability of 1 m^2^/ewe as compared to 2 to 3 m^2^/ewe. Shorter and sinuous trajectories also resulted in lower net distances at the lowest space availability (defined as the straight distance from the departing to the final location), effect that was close to significance. The restriction in movements when space was most limited could be explained by reduced dimensions of the enclosure or by the barrier effect caused by the presence of other ewes encountered in the path of movement, as described for other production species [36], [52]. The frequent encounter of walls or of individuals in the path of movement either forced the ewes to change direction or negotiate an alternative path around other individuals, resulting in increased sinuosity of the trajectory, or to stop altogether if motivation to move was low. These findings are in agreement with the reduction in the total and net distances also described for broiler chickens housed at varying stocking densities and enclosure sizes [31], [52]. The idea that the limitation in the ‘freedom of movements’ can be due to a barrier effect likely due to the close proximity of pen mates, which hindered the free movement of ewes within the enclosure, appeared to be further supported by the shorter maximum step length detected when space was limited to 1 m^2^/ewe, evidencing that limitation can be already perceived in short term movements.

The effects related to high density and those of reduced enclosure size are difficult to distinguish. Leone and Estevez [31] systematically controlled for the effects of density *versus* those of enclosure size, finding that total distance appeared to be more related to density, while net distance was associated with enclosure dimensions. This might have happened in the present study as well. However, it is noteworthy and surprising the fact that, contrary to the results obtained in chickens, in this study no differences were detected across space treatments of 2 and 3 m^2^/ewe regarding travelled distance or in terms of mean inter-individual distances. A plausible explanation for this may reside in the long inactive periods characterizing ewes in advanced gestation, with ewes spending over 70% of their time resting or in idle standing positions [18]. On the other hand, when looking at the nearest and furthest neighbour distances, these increased progressively as space availability increased from 1 to 3 m^2^/ewe, suggesting that inter-individual spacing is adjusted according to the dimensions of the enclosure, even if group size remains constant. In addition, the lower movement activity rate would also support that lack of differences in movement patterns for ewes housed at both 2 and 3 m^2^/ewe, as compared to 1 m^2^/ewe could be due to the longer undisturbed resting periods at the higher space availabilities. All these results provide evidences that limiting space availability to 1 m^2^/ewe compromises length, trajectory patterns, and inter-individual distances in gestating ewes.

The increase in both nearest and furthest neighbour distances with space availability would agree with results found for domestic fowls [31] and grazing sheep, in which a reduction in nearest neighbour distances when decreasing space availability from 200 to 50 m^2^/head was observed [46]. Results would reflect ewes' tendency to maximize inter-individual distances when given the opportunity, perhaps to minimize resource competition [30], [53]. Inter-individual distances in sheep depend on their degree of activity within the flock [45], so that increase in both nearest and furthest neighbour distances as space availability increased would reflect higher activity levels, as already observed in chickens [52]. We indeed observed that ewes initiated fewer movements and spent a smaller proportion of time moving at 1 m^2^/ewe regarding the other treatments, as well as an increase in the frequency of social interactions [18], confirming that the ability to move was altered under the most severe space restriction conditions. Contrarily, increased inter-individual distances occurred from 2 to 3 m^2^/ewe, even though no differences were detected in total and net distance or step length.

It is important to highlight that movement activity was higher at 1 m^2^/ewe, indicating more frequent changes in location within the enclosure. Taking into account that ewes at the lowest space availability were closer to each other and tended to spend less time resting [18], it may be suggested that ewes housed at the lowest space availability experienced more disturbances during the resting periods and increased the restlessness levels, similar to the effects found in chickens [52]. The higher number of disturbances would lead to changes in position, what may also explain the higher frequency of social interactions, both positive and negative, found at 1 m^2^/ewe [18].

Ewes al the lowest space availability also showed a higher preference for locations next to the wall as compared to larger space availability. However, the space treatment effect disappeared when values were corrected according to the expected values. Nevertheless, the observed wall locations were chosen more frequently than expected, reflecting an overall preference of ewes for enclosure areas next to the walls, as previously observed for chicken [38] and for sheep [54], where ewes spend most of their lying time [19]. This preference might be explained because animals perceive walls as a safer, more protected area from potential predators or from the interactions with other conspecifics. No attempt was done in this study to determine the effect of space allowance on the use of space according to the activity of ewes, although further information regarding this aspect would help to acquire a better understanding about the group dynamics of ewes under spatial restriction conditions.

### Gestation week

Only minor effects were observed as the experiment progressed, mostly related to the higher movement activity detected during the first week of study. Total travelled distance decreasing after week 1 would be likely due to both the disappearance of the novelty effect that promoted a higher expression of exploratory behaviours [18], and to the normalization of the social dynamics created after introducing the ewes into a confined space. Novelty may be a stressful experience for animals [55], and longer total travelled distances during week 1 would be explained by restlessness and exploration of the new environment. Net to total distance ratio was also lower during week 1, indicating higher path sinuosity during the adaptation period, what would be mainly due the predominant explorative behaviours during this period, as described in our simultaneous study [18], where the subsequent reduction in the frequency of exploring was particularly apparent at 1 m^2^/ewe. Increased angular dispersion values from week 2 until the end of the experiment would also agree with net to total distance ratio findings, suggesting that trajectories became less sinuous.

Although maximum step length decreased after week 1, variation between week 2 and 8 was high, with values only becoming consistently lower than initial ones at week 9. This might be a consequence of the development of pregnancy, in the sense that from week 9 to the end of the study ewes became very heavy and voluminous, making shorter steps when moving. Minimum step length dropped after week 1, suggesting a decrease in the number of movements within the pen after initial adaptation that would be confirmed by the reduction in movement activity from week 2 until the end of the experiment. The increase in nearest neighbour distances as the lambing period approached (week 11) with respect to initial values might reflect ewes' willingness to isolate from the rest of the flock [56]. Real isolation from the group was actually not possible though, and consequently the increase in nearest neighbour distances would most likely be a consequence of the substantial increase in ewes' body volume similar to the effects of growth found for chickens [31]. An interaction between space availability and gestation week was expected as consequence of the exacerbation of the spatial restriction as pregnancy advanced. No interactions were detected, suggesting that spatial requirements for ewes are not modified by the natural changes in body size occurring through pregnancy.

In conclusion, results of this study indicate that the reduction in space availability to 1 m^2^/ewe limited the movement of confined pregnant ewes. This restriction was evidenced by shorter and more sinuous trajectories composed of shorter steps, lower inter-individual distances and higher movement activity, the latter potentially linked to higher restlessness levels. On the other hand, the limited differences between 2 and 3 m^2^/ewe related only to minor increments in inter-individual distances that would likely be explained by the longer resting and inactive periods. Therefore, under the conditions of the present experiment, it would appear that increasing space availability from 2 to 3 m^2^/ewe would result in limited benefits during gestation. In addition, through gestation only small variations in movement patterns were detected, mainly restricted to slight increments in nearest and furthest neighbour distances, suggesting that spatial requirements for sheep remained stable through the gestation period.

## References

[1] Morand-Fehr P, Fedele V, Decandia M, Le Frileux Y (2007) Influence of farming and feeding systems on composition and quality of goat and sheep milk. Small Rum Res 68: 20–34.

[2] Waterhouse A (1996) Animal welfare and sustainability of production under extensive conditions – A European perspective. Appl Anim Behav Sci 49: 29–40.

[3] Dwyer C (2004) How has the risk of predation shaped the behavioural responses of sheep to fear and distress? Anim. Welfare 13: 269–281.

[4] Petherick JC (2007) Spatial requirements of animals: Allometry and beyond. J Vet Behav 2: 197–204.

[5] Brambell Committee (1965). Report of the Technical Committee to enquire into the welfare of animals kept under intensive livestock husbandry systems. Command paper 2836 . London, UK: Her Majesty's Stationery Office.

[6] Averós X, Brossard L, Dourmad JY, de Greef KH, Edge HL, et al (2010) Quantitative assessment of the effects of space allowance, group size and floor characteristics on the lying behaviour of growing-finishing pigs. Animal 4: 777–783. 22444133 10.1017/S1751731109991613

[7] Averós X, Brossard L, Dourmad JY, de Greef KH, Edge HL, et al (2010) A meta-analysis of the combined effect of housing and environmental enrichment characteristics on the behaviour and performance of pigs. Appl Anim Behav Sci 127: 73–85.

[8] Averós X, Brossard L, Dourmad JY, de Greef KH, Edwards SA, et al (2012) Meta-analysis on the effects of the physical environment, animal traits, feeder and feed characteristics on the feeding behaviour and performance of growing-finishing pigs. Animal 6: 1275–1289. 23217231 10.1017/S1751731112000328

[9] Turner SP, Ewen M, Rooke JA, Edwards SA (2000) The effect of space allowance on performance, aggression and immune competence of growing pigs housed on straw deep-litter at different group sizes. Livest Prod Sci 66: 47–55.

[10] Gonyou HW, Brumm MC, Bush E, Deen J, Edwards SA, et al (2006) Application of broken-line analysis to assess floor space requirements of nursery and grower-finisher pigs expressed on an allometric basis. J Anim Sci 84: 229–235. 16361511 10.2527/2006.841229x

[11] Ingvartsen KL, Andersen HR (1993) Space allowance and type of housing for growing cattle: A review of performance and possible relation to neuroendocrine function. Acta Agr Scand A – Anim Sci 43: 65–80.

[12] Krawczel PD, Klaiber LB, Butzler RE, Klaiber LM, Dann HM, et al (2012) Short-term increases in stocking density affect the lying and social behaviour, but not the productivity, of lactating Holstein dairy cows. J Dairy Sci 95: 4298–4308. 22818444 10.3168/jds.2011-4687

[13] Wechsler B (2011) Floor quality and space allowance in intensive beef production: A review. Anim Welfare 20: 497–501.

[14] Bessei W (2006) Welfare of broilers: a review. World Poult Sci J 62: 455–466.

[15] Cornetto T, Estevez I, Douglass LW (2002) Using artificial cover to reduce aggression and disturbances in domestic fowl. Appl Anim Behav Sci 75: 325–336.

[16] Estevez I (2007) Density allowances for broilers: where to set the limits? Poult Sci 86: 1267–1272. 10.1093/ps/86.6.1265 17495104

[17] Lay DC Jr, Fulton RM, Hester PY, Karcher DM, Kjaer JB, et al (2011) Hen welfare in different housing systems. Poult Sci 90: 278–294. 21177469 10.3382/ps.2010-00962

[18] Averós X, Lorea A, Beltrán de Heredia I, Ruiz R, Marchewka J, et al (2014) The behaviour of gestating dairy ewes under different space allowances. Appl Anim Behav Sci 150: 17–26.

[19] Bøe KE, Berg S, Andersen IL (2006) Resting behaviour and displacement in ewes-effects of reducing lying space and pen shape. Appl Anim Behav Sci 98: 249–259.

[20] Sevi A, Massa S, Annicchiarico G, Dell'aquila S, Muscio A (1999) Effect of stocking density on ewes milk yield, udder health and microenvironment. J Dairy Res 66: 489–499. 10612048 10.1017/s0022029999003726

[21] Arehart LA, Lewis JM, Hinds FC, Mansfield ME (1969) Space allowance for lambs on slotted floors. J Anim Sci 29: 638–664.

[22] Gonyou HW, Stookey JM, McNeal LG (1985) Effects of double decking and space allowance on the performance and behaviour of feeder lambs. J Anim Sci 60: 1110–1116. 4008359 10.2527/jas1985.6051110x

[23] Horton GMJ, Malinowski K, Burgher CC, Palatini DD (1991) The effect of space allowance and sex on blood catecholamines and cortisol, feed consumption and average daily gain in growing lambs. Appl Anim Behav Sci 32: 197–204.

[24] Fraser AF, Broom DM (1997) Farm Animal Behaviour and Welfare. 3rd edition. Wallingford, Oxfordshire, UK: CAB International. 437 p.

[25] Gonyou HW (1991) Behavioral methods to answer questions about sheep. J Anim Sci 69: 4155–4160. 1778830 10.2527/1991.69104155x

[26] Keeling L (1995) Spacing behaviour and an ethological approach to assessing optimum space allocations for groups of laying hens. Appl Anim Behav Sci 44: 171–186.

[27] Dwyer C (2009) The behaviour of sheep and goats. In: Jensen P, editor. The Ethology of Domestic Animals, 2^nd^ Edition: An Introductory Text.Wallingford, Oxfordshire, UK: CAB International. pp. 161–176.

[28] Grigor PN, Hughes BO, Appleby MC (1995) Emergence and dispersal behaviour in domestic hens: effects of social rank and novelty of an outdoor area. Appl Anim Behav Sci 45: 97–108.

[29] Gueron S, Levin SA, Rubenstein DI (1996) The dynamics of herds: from individuals to aggregations. J Theor Biol 182: 85–98.

[30] Leone EH, Estevez I (2008) Space use according to the distribution of resources and level of competition. Poult Sci 87: 3–13. 18079443 10.3382/ps.2007-00026

[31] Leone EH, Estevez I (2008) Use of space in the domestic fowl: separating the effects of enclosure size, group size and density. Anim Behav 76: 1673–1682.

[32] Arnold GW, Maller RA (1985) An analysis of factors influencing spatial-distribution in flocks of grazing sheep. Appl Anim Behav Sci 14: 173–189.

[33] Warburton K, Lazarus J (1991) Tendency distance models of social cohesion in animal groups. J Theor Biol 150: , 473–488. 10.1016/s0022-5193(05)80441-2 1943130

[34] Beecham JA, Farnsworth KD (1999) Animal group forces resulting from predator avoidance and competition minimisation. J Theor Biol 198: , 533–548. 10.1006/jtbi.1999.0930 10373353

[35] Dove H, Beilharz RG, Black JL (1974) Dominance patterns and positional behaviour of sheep in yards. Anim Prod 19: 157–168.

[36] Newberry RC, Hall JW (1990) Use of pen space by broiler chickens: effects of age and pen size. Appl Anim Behav Sci 25: 125–136.

[37] Buijs S, Keeling LJ, Vangestel C, Baert J, Tuyttens FAM (2011) Neighbourhood analysis as an indicator of spatial requirements of broiler chickens. Appl Anim Behav Sci 129: 111–120.

[38] Leone EH, Christman MC, Douglass L, Estevez I (2010) Separating the impact of group size, density, and enclosure size on broiler movement and space use at decreasing perimeter to area ratio. Behav Process 83: 16–22. 10.1016/j.beproc.2009.08.009 19733638

[39] Mallapur A, Miller C, Christman MC, Estevez I (2009) Short-term and long-term movement patterns in confined environments by domestic fowl: influence of group size and enclosure size. Appl Anim Behav Sci 117: 28–34.

[40] Buijs S, Keeling LJ, Vangestel C, Baert J, Vangeyte J, et al (2010) Resting or hiding? Why broiler chickens stay near walls and how density affects this. Appl Anim Behav Sci 124: 97–103.

[41] Cornetto T, Estevez I (2001) Influence of vertical panels on use of space by domestic fowl. Appl Anim Behav Sci 71: 141–153. 11179566 10.1016/s0168-1591(00)00171-4

[42] Buijs S, Keeling LJ, Vangestel C, Baert J, Vangeyte J, et al (2011) Assessing attraction or avoidance between rabbits: Comparison of distance-based methods to analyse spatial distribution. Anim Behav 82: 1235–1243.

[43] Turner SP, Nath M, Horgan GW, Edwards SA (2013) Measuring chronic social tension in groups of growing pigs using inter-individual distances. Appl Anim Behav Sci 146: 26–36.

[44] Michelena P, Bouquet PM, Dissac A, Fourcassie V, Lauga J, et al (2004) An experimental test of hypotheses explaining social segregation in dimorphic ungulates. Anim Behav 68: 1371–1380.

[45] Michelena P, Gautrais J, Gérard J-F, Bon R, Deneubourg J–L (2008) Social cohesion in groups of sheep: effect of activity level, sex composition and group size. Appl Anim Behav Sci 112: 81–93.

[46] Sibbald AM, Shellard LJF, Smart TS (2000) Effects of space allowance on the grazing behaviour of sheep. Appl Anim Behav Sci 70: 49–62. 10986423 10.1016/s0168-1591(00)00145-3

[47] Vandenheede M, Bouissou MF (1993) Sex differences in fear reactions in sheep. Appl Anim Behav Sci 37: 39–55.

[48] Russel AJF, Doney JM, Gunn RG (1969) Subjective assessment of body fat in live sheep. J Agric Sci 72: 451–454.

[49] Sanchez C, Estevez I (1998) The Chickitizer Software Program. College Park, Maryland, USA: University of Maryland.

[50] Turchin P (1998) Quantitative Analysis Movement: Measuring and Modelling Population Redistribution in Animals and Plants. Sunderland, MA, USA: Sinauer Associates. 396 p.

[51] Estevez I, Mallapur A, Miller C, Christman MC (2010) Short- and long-term movement patterns in complex confined environments in broiler chickens: the effects of distribution of cover panels and food resources. Poult Sci 89: 643–650. 20308395 10.3382/ps.2009-00433

[52] Estevez I, Newberry RC, Arias de Reyna L (1997) Broiler chickens: A tolerant social system? Etologia 5: 19–29.

[53] Stahl J, Tolsma PH, Loonen MJJE, Drent RH (2001) Subordinates explore but dominants profit: Resource competition in high Arctic barnacle goose flocks. Anim Behav 61: 257–264. 11170715 10.1006/anbe.2000.1564

[54] Marsden MD, Wood-Gush DGM (1986) The use of space by group-housed sheep. Appl Anim Behav Sci 15: 178.

[55] Dantzer R, Mormède P (1983) Stress in farm animals: A need for reevaluation. J Anim Sci 57: 6–18. 6350254 10.2527/jas1983.5716

[56] Pollard JC (2006) Shelter for lambing sheep in New Zealand: A review. New Zeal J Agric Res 49: 395–404.

